# An *Ex Vivo* Model in Human Femoral Heads for Histopathological Study and Resonance Frequency Analysis of Dental Implant Primary Stability

**DOI:** 10.1155/2014/535929

**Published:** 2014-06-04

**Authors:** Pedro Hernández-Cortés, Alberto Monje, Pablo Galindo-Moreno, Andrés Catena, Inmaculada Ortega-Oller, José Salas-Pérez, Francisco Mesa, Rafael Gómez-Sánchez, Mariano Aguilar, David Aguilar, Francisco O'Valle

**Affiliations:** ^1^Department of Traumatology and Orthopedic Surgery, “San Cecilio” Clinical Hospital, University of Granada, Spain; ^2^Department of Periodontics and Oral Medicine, School of Dentistry, University of Michigan, Ann Arbor, MI, USA; ^3^Oral Surgery and Implant Dentistry Department, School of Dentistry, University of Granada, Granada, Spain; ^4^Department of Experimental Psychology, School of Psychology, University of Granada, Granada, Spain; ^5^Department of Periodontics, School of Dentistry, University of Granada, Granada, Spain; ^6^Department of Pathology, School of Medicine and Institute of Biopathology and Regenerative Medicine (IBIMER), University of Granada, Granada, Spain; ^7^Departamento de Anatomía Patológica, Facultad de Medicina, 18012 Granada, Spain

## Abstract

*Objective*. This study was designed to explore relationships of resonance frequency analysis (RFA)—assessed implant stability (ISQ values) with bone morphometric parameters and bone quality in an *ex vivo* model of dental implants placed in human femoral heads and to evaluate the usefulness of this model for dental implant studies. *Material and Methods*. This *ex vivo* study included femoral heads from 17 patients undergoing surgery for femoral neck fracture due to osteoporosis (OP) (*n* = 7) or for total prosthesis joint replacement due to severe hip osteoarthrosis (OA) (*n* = 10). Sixty 4.5 × 13 mm Dentsply Astra implants were placed, followed by RFA. CD44 immunohistochemical analysis for osteocytes was also carried out. *Results*. As expected, the analysis yielded significant effects of femoral head type (OA versus OA) (*P* < 0.001), but not of the implants (*P* = 0.455) or of the interaction of the two factors (*P* = 0.848). Bonferroni post hoc comparisons showed a lower mean ISQ for implants in decalcified (50.33 ± 2.92) heads than in fresh (66.93 ± 1.10) or fixated (70.77 ± 1.32) heads (both *P* < 0.001). The ISQ score (fresh) was significantly higher for those in OA (73.52 ± 1.92) versus OP (67.13 ± 1.09) heads. However, mixed linear analysis showed no significant association between ISQ scores and morphologic or histomorphometric results (*P* > 0.5 in all cases), and no significant differences in ISQ values were found as a function of the length or area of the cortical layer (both *P* > 0.08). *Conclusion*. Although RFA-determined ISQ values are not correlated with morphometric parameters, they can discriminate bone quality (OP versus OA). This *ex vivo* model is useful for dental implant studies.

## 1. Introduction

Primary implant stability, which is essential for osseointegration [1, 2] and the success of implant therapy [3], is influenced by bone quality and quantity, implant design, and drilling protocol [4]. A quantitative measurement of bone quality is therefore an essential component of dental implantation planning.

Many methods have been proposed to assess initial implant stability, but most of them are no longer used due to their invasiveness and inaccuracy [5]. Primary implant stability is most frequently determined by using cutting-torque measurements or resonance frequency analysis (RFA) [6], which evaluates the micromotion or displacement of the implant in bone under a lateral load, applying microscopic lateral forces to the implant with a vibrating transducer [7, 8]. Results are given as implant stability quotients (ISQs) [9], which are affected by three main factors: the stiffness of the implant fixture and its interface with surrounding tissue, the design of the transducer, and the total effective implant length above bone level [10]. ISQs range from 0 to 100, with higher number indicating greater stability. No definitive threshold value has been established to differentiate a stable, integrated implant from a failing/failed implant; however, it has been suggested that an ISQ value above 57 at 1 year after loading represents a successful implant outcome [11], with a value below 50 indicating a risk of implant failure [12].

Various* in vivo* studies have been performed on the reliability of RFA to predict implant success, on the influence of bone quality on ISQ values, and on a cut-off point to predict implant failure [11, 13–16]. In a previous study by our group, ISQ values showed a low sensitivity and did not reliably predict early implant failure, and no cut-off value could be established for differentiating between success and early failure [16]. The objective of the present study was to explore the relationships of RFA ISQ values for standardized implants with bone morphometric parameters and bone quality in an* ex vivo* model of dental implants placed in human femoral heads with different trabecular bone qualities, in an evaluation of the usefulness of this model for dental implant studies

## 2. Material and Methods 

This* ex vivo* study used femoral heads from 17 patients undergoing surgery in the Orthopedic Surgery Department of our hospital for either femoral neck fracture due to osteoporosis (*n* = 7) or total prosthesis joint replacement due to severe hip osteoarthrosis (*n* = 10). Informed consent was obtained from all patients before the treatment, and the study was independently reviewed and approved by the local ethical committee of our institution.

### 2.1. Implant Placement

**Figure 1:**
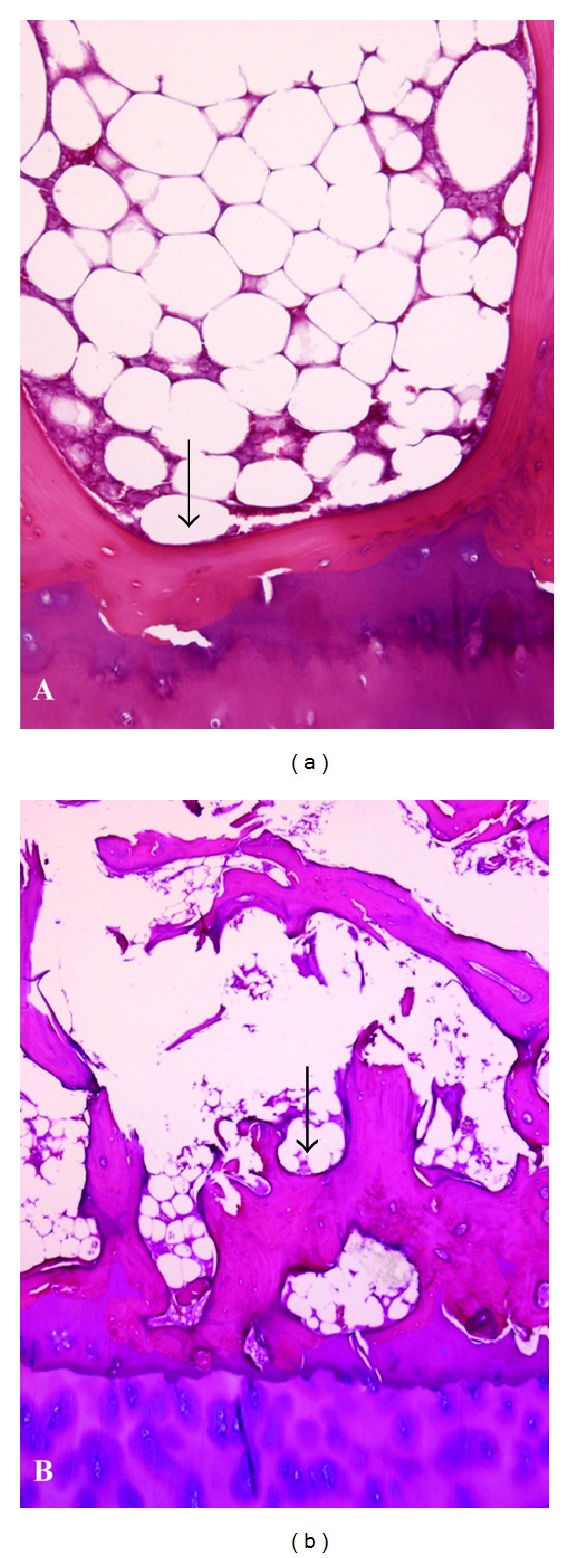
Femoral head cortical area. Note the different area between osteoporosis (a) and osteoarthrosis (b) in 3 mm diameter trephine biopsies. Narrow: different thickness of subchondral bone.

Sixty 4.5 × 13 mm implants (Dentsply Astra implants, Mölndal, Sweden) were placed immediately after femoral head extraction (4 implants in each femoral head with interimplant distance of 5 mm). Implant placement and drilling protocol were performed for conventional sockets (starting with a round bur and ending with a 4.5 mm twister bur) following the manufacture's recommendations and applying the same final torque (40 N/cm) in all cases (Figure 1).

### 2.2. Resonance Frequency Analysis (RFA)

An Osstell ISQ RFA device (Integration Diagnostics AB, Göteborg, Sweden) was used to measure primary implant stability according to the manufacturer's recommendations. Briefly, a metal rod (SmartPeg, Integration Diagnostics AB, Göteborg, Sweden) was screwed at a torque of 40 N/cm into the implant screw vent. Three measurements were performed for each implant: one parallel to the long axis of the implant and two perpendicular to this axis in two different positions on the transducer. These measurements were performed three times during the study period: immediately after epiphysis extraction (fresh), after 72 h of fixation in 10% buffered formalin (fixated), and at 20 days after fixation (decalcified).

### 2.3. Sample Biopsies

Four sample biopsies were obtained from each femoral head using trephines with external diameter of 3 mm and internal diameter of 2 mm, producing a total of 68 trephine core biopsies, 28 from osteoporotic (OP) and 40 from osteoarthrotic (AO) femoral heads. An additional sample was taken from each femoral head using trephines with external diameter of 5 mm and internal diameter of 4 mm (*n* = 17).

### 2.4. Morphologic and Histomorphometric Analysis

Biopsy samples were fixed in 10% buffered formalin for 72 h. Samples then were decalcified at room temperature in 10% formaldehyde, 8% formic acid, and 1% methanol (Decalcifier I, Surgipath Europe Ltd., Peterborough, UK), for 20 days. Next, samples were embedded in paraffin. When required, 4 *μ*m sections were cut along the long axis of the biopsy, dewaxed, and rehydrated for staining with hematoxylin-eosin, periodic acid Schiff, and Masson's trichrome.

Histomorphometric evaluation was carried out using a light microscope BX51 (Olympus Optical Company, Ltd., Tokyo, Japan) equipped with a high resolution video camera (3CCD, DP70, Olympus) connected to a monitor and PC (Intel Core2, Intel, Santa Clara, CA), using the ImageJ 1.47 histomorphometric software package (NIH, http://rsb.info.nih.gov/ij/).

Ten images (10x) of H&E staining per core were captured using fluorescence light. Image normalization and automatic thresholding were used to obtain binary images for measuring the area, circumference, and number of bone particles. The subchondral bone thickness was also evaluated, performing five measurements to obtain mean, maximum, and minimum thickness values.

### 2.5. CD44 Immunohistochemical Analysis

For the immunohistochemical study, biopsies were dewaxed and then unmasked for antigen retrieval in ethylenediaminetetraacetic acid (EDTA) buffer solution (pH8) at 95°C for 20 min in a PT module (Thermo Fisher Scientific, Kalamazoo, MI, USA). Once tempered, all slides were introduced into an automatic immunostainer (Autostainer480, Thermo Fisher Scientific) using the two-step micropolymer-peroxidase-based method (Ultravision Quanto, Thermo Fisher Scientific), followed by development with diaminobenzidine. Bone sections were incubated for 10 min with prediluted monoclonal anti-CD44 (clone: 156-3C11). The signal was amplified by incubating for 10 min with amplifier antibody and for a further 10 min with micropolymer conjugated with peroxidase. All reagents were acquired from Master Diagnóstica (Granada, Spain).

A millimeter scale in the eyepiece of a BH2 microscope (Olympus Optical Company, Ltd.) with a 40x objective was used to count the number of CD44-positive osteocytes per mm^2^; this number was then divided by 0.062 (correction value for 40x magnification).

### 2.6. Statistical Analysis

The mixed linear model, implemented in SPSS version 20.0 (IBM SPSS Inc., Chicago, IL), was used to analyze differences in ISQs as a function of implants and femoral bone treatment (fresh, fixed, or decalcified). A diagonal repeated-measures covariance structure was developed following Schwarz's Bayesian criterion. This model was also used to analyze the effects of bone type (OP versus OA) on ISQ scores and to disentangle the effects of bone type on morphologic features. Bonferroni corrected comparisons were done when necessary. *P* < 0.05 was considered significant.

## 3. Results

**Figure 2:**
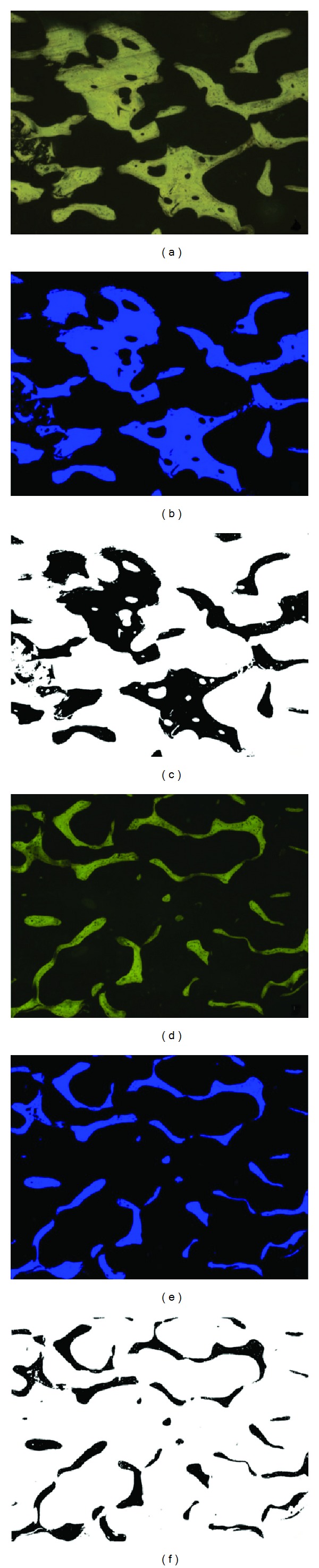
Image analysis process. (a) Osteoarthrosis femoral head sample. (b) Osteoporosis femoral head sample. Note the following: (a) and (d) are trabecular area (yellow) hematoxylin-eosin stained and visualized with fluorescence microscopy, (b) and (e) are threshold images, and (c) and (f) are binary images with interest area in black.

The morphometric study showed a difference in trabecular bone quality between OP and OA femoral heads. In comparison to the OA heads, the subchondral bone was significantly thinner (303.1 ± 59.1 versus 716.1 ± 228.4 square micrometers, *P* = 0.002 Bonferroni test), and the subchondral bone area was significantly smaller (3.41 ± 5.2 versus 12.61 ± 7.5 mm^2^, *P* = 0.04 Bonferroni test) in the OP group (Figure 1). The trabecular bone area was also lower (0.29 ± 0.02 versus 0.38 ± 0.02 mm^2^, *P* = 0.004), with greater bone fragmentation (16.71 ± 1.29 versus 12.83 ± 1.14   *n*° particles, *P* = 0.028), in the OP* versus* OA femoral heads (Figure 2).

**Figure 3:**
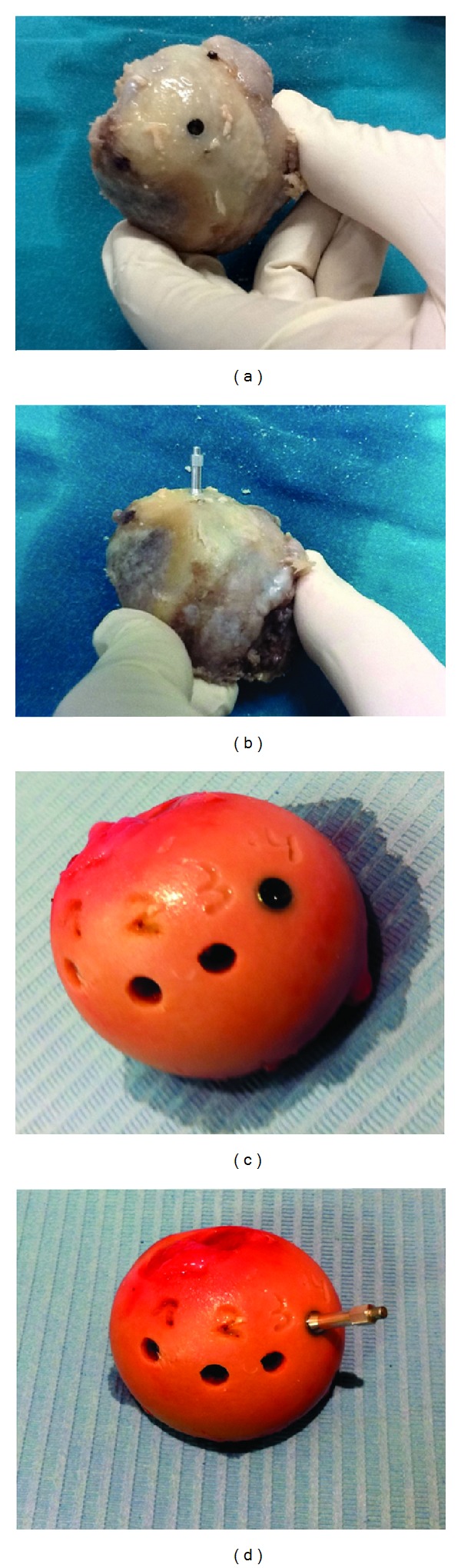
Fresh femoral head with coxarthrosis ((a) and (b)) and osteoporosis ((c) and (d)). (a) Frontal view of dental implant. (b) Dental implant with the SmartPeg placed for determination of implant primary stability in terms of implant stability quotients (ISQ value).

In this* ex vivo* model (Figure 3), high primary stability (ISQ > 70) was obtained in 65% (26/40) of dental implants placed in OA heads but in only 28.6% (8/28) of those placed in OP heads. Low primary stability (ISQ values <60) was observed in 10% (4/40) of implants in OA and 7.1% (2/28) of those in OP heads. Despite the significantly greater thickness and area of OA bone samples, no significant correlation was found between ISQ values and the length or area of the cortical layer (both *P* > 0.08). However, there was a tendency towards higher ISQ values in implants in OA heads.

### 3.1. ISQ Values Obtained from Fresh versus Fixed versus Decalcified Femoral Heads

**Figure 4:**
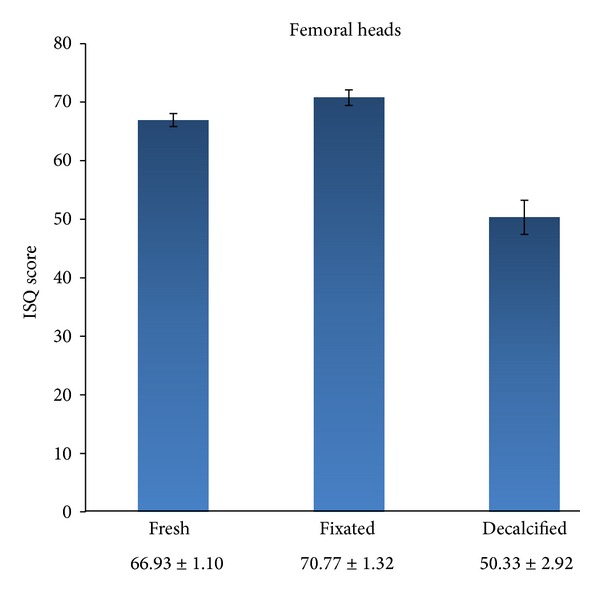
Comparison of ISQ values of dental implants in femoral heads under different physicochemical conditions.

The biomechanical properties of the femoral heads were modified by the fixation and decalcification processes (treatments). The analysis yielded significant effects on ISQ values of femoral head treatment, *F* (2, 54.112) = 20.84, *P* < 0.001, but not of the implants, *F* (3, 41.175) = 0.889, *P* = 0.455, or of the interaction of both factors, *F* (6, 33.296) = 0.439, *P* = 0.848. Bonferroni post hoc comparisons demonstrated lower mean ISQ values for implants in decalcified (50.33 ± 2.92) than in fresh (66.93 ± 1.10) or fixated (70.77 ± 1.32) heads (both *P* < 0.001) (Figure 4). No other significant differences were observed (Table 1).

### 3.2. ISQ Values from the OA versus OP Femoral Head Specimen

The mean ISQ score was higher for implants in OA (73.52 ± 1.92)* versus* OP (67.13 ± 1.09) heads, *F* (1, 63) = 15.229, *P* < 0.001. When the mean ISQ value for each treatment group (fresh, fixated or calcified) was considered, no significant effect was found for bone type, *F* (1, 40.63) = 0.068, *P* = 0.796, or for the interaction of bone pathology with femur treatment (fresh versus decalcified), *F* (1, 40.63) = 1.841, *P* = 0.182, although significant differences in ISQ values were observed as a function of femoral head treatment, *F* (1, 40.63) = 39.42, *P* < 0.01.

### 3.3. ISQ Values and Morphologic and Histomorphometric Values

Mixed linear analysis showed no significant association between ISQ scores and morphologic or histomorphometric results (*P* > 0.5 in all cases).

### 3.4. ISQ Values and Immunohistochemical Results

**Figure 5:**
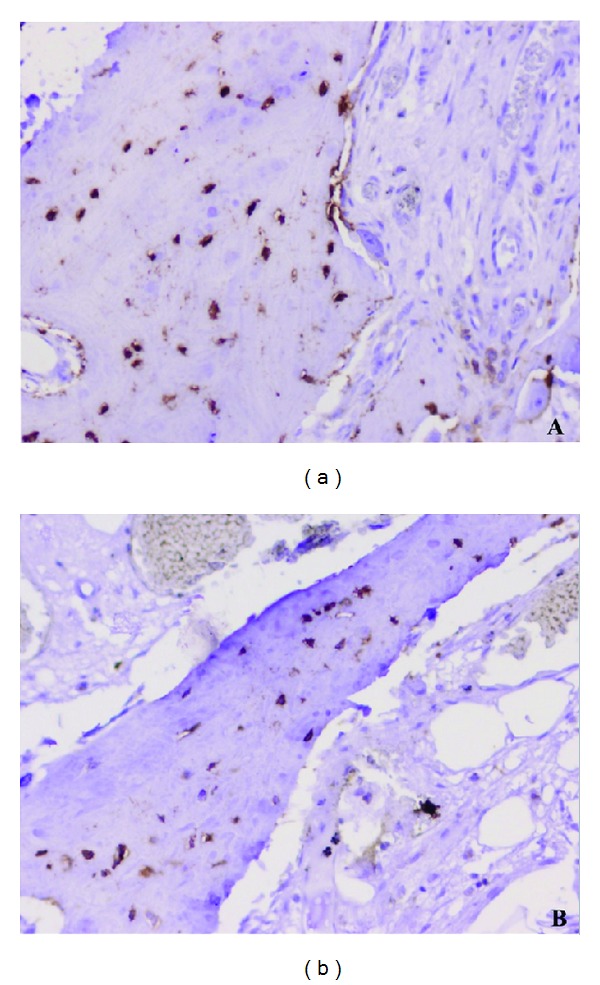
CD44-positive osteocytes in trabecular bone. (a) Femoral head with osteoarthrosis. (b) Femoral head with osteoporosis (micropolymer-peroxidase-based method, original magnification ×20).

Linear correlation analysis showed no significant correlation between ISQ scores and CD44 count (*r* = −0.025, *P* < 0.472). The number of osteocytes per mm^2^ in trabecular bone was the same (*P* = 0.470) between OP (135.74 ± 40.19) and OA (147.17 ± 47.59) samples (Figure 5).

## 4. Discussion

This study explored the relationship between RFA ISQ values and bone morphometric parameters or bone quality in an* ex vivo* model of dental implants placed in human femoral heads. This model reduces the risk of bias due to external factors and avoids the limitations of* in vivo* research.

Several studies [17–19] have attempted to establish correlations between bone-to-implant contact (BIC) and ISQ values. One study of the sensitivity of ISQ values to detect early implant failure, based on immediate postimplant values, reported 73.7% correct classifications, with an incorrect classification of 55% of implant failures, whereas 86.2% correct classifications were obtained with delayed (4-month) ISQ values at the cost of assuming the survival of all implants [16].

Experimental and clinical studies have shown bone density to be a major determinant of RFA-assessed primary stability after implant placement, supporting observations by Meredith and coworkers [20]. Thus, RFA results were found to correlate with insertion torque measurements [21, 22], clinically-assessed bone density [23], and CT-assessed bone density (in Hounsfield units) [21, 24]. Some studies have demonstrated a relationship between bone density and implant primary stability* in vivo* [25–27], while others have used* ex vivo *models in cadaver bone [28] and femoral heads of swine [29]. However, there are difficulties in extrapolating results obtained in animals to humans, and lower bone density values can be expected in dry cadaver bone than in fresh vital bone [28]. The present study aimed to overcome these limitations by using fresh human femoral heads, the same drilling protocol for implant placement, and the same implant system, minimizing biases and obtaining more accurate data on the relationship between ISQ values and peri-implant bone.

To our best knowledge, only one study has used human femoral heads to determine implant stability by RFA [6]; it reported a slightly lower mean ISQ (59.3 ± 2.4) than in the present study, possibly because they derived the heads from frozen cadavers. A further factor may be the smaller diameter of the implants used in our study, because ISQ values have been shown to be influenced by implant diameter [30].

Biomechanical conditions can be changed by fixation and decalcification processes. Formalin fixation destroys the three-dimensional conformation of proteins by cross-linking their structure, producing a loss of elasticity in bone [31], although it has been reported that short-term formalin fixation does not affect its mechanical characteristics [32]. It is not yet clear how fixation with formalin influences ISQ values and the mechanical characteristics of bone. However, Morita and coworkers, using a rabbit tibia model, observed that formalin fixation appears to affect bone mechanical characteristics by binding amino-proteins and forming bridges between the formalin and protein, which potentially influences three-dimensional protein structure, whereas it does not appear to influence ISQ values [33].

The present results are in agreement with previous reports on the relationship between ISQ values and bone density by localization [5, 14, 34–39]. The osteoporotic bone in our study was bone type III-IV and the osteoarthrosis bone was type I-II according to Lekholm and Zarb bone classification [40]. Although the osteoporotic bone biopsy samples felt more fragile, no significant difference in ISQ values was found between the OA and OP bone, although this may be explained by the small sample size. This limitation may also explain the lack of significant difference in ISQ values between the bone types as a function of morphologic or histomorphometric results, given that bone density is known to be much lower in patients with osteoporosis [41]. These results suggest that the ISQ value is not determined by a specific amount of trabeculae but rather by the cortical thickness around the implant neck [39, 40]. In a recent* ex vivo* study in human cadavers, Roze et al. [42] found that ISQ values differed among cortical, mixed, and cancellous types of bone. They reported that ISQ values were significantly correlated with cortical thickness but not with bone histomorphometric parameters. Cehreli et al. [6], using the same model as in the present study, also found that ISQ values in fresh femoral heads were influenced by the cortical bone thickness. In fact, the discrepancy in ISQ values with the present study may be related to the thickness values, which were less uniform in our series. Although the trabecular bone thickness may play a minimal role in implant primary stability, it is of major importance in peri-implant bone healing [39, 42]. In disagreement with previous findings, our results show that the utilization of OP and OA femoral heads offers a feasible model to determine safely the relationship between ISQ values and cortical layer thickness (*P* = 0.04). However, no significant difference was found between OP and OA heads when correlated with the cortical layer (*P* = 0.08). Hence, despite a tendency towards increased ISQ values in OA heads, the results do not offer sufficient reliability for selecting the appropriate implant loading protocol based on implant primary stability.

ISQ values were lower in decalcified samples than in fresh or fixed samples (*P* < 0.001), supporting the use of formalin-fixed samples as a means of extending the time frame for their study. ISQ values obtained by Cömert et al. [43] in fresh-frozen human bone were similar to those in formalin-fixed bone, and the preservation of the latter's biomechanical properties makes it appropriate for the study of primary stability. We highlight that the significantly lower ISQ values in decalcified samples indicate the involvement of calcium in RFA-assessed primary stability.

We counted the number of osteoblastic (CD44-positive) cells as a quantitative measure of bone quality. Osteocytes, which represent 95% of bone cells, are old osteoblasts that fill lacunae through a network of tubules connected to the outer surface of the trabeculae [44]. The lack of a significant difference between OA and OA bone in the present study suggests that the CD44 count is not a reliable indicator of bone quality (*r* = −0.025,   *P* = 0.472).

## 5. Conclusion

It is possible to discriminate bone quality (osteoporotic versus osteoarthrosis) by using RFA (ISQs), and the* ex vivo* model described here is useful for dental implant-related studies. However, no correlation was found between RFA-assessed primary implant stability and trabecular bone structures or cortical layer thickness. Further studies with larger sample sizes are needed to elucidate the influence of cortical thicknesses on primary implant stability and the role played by the trabecular structure in implant stability after the healing process.

## Figures and Tables

**Table 1:** Comparison of ISQ values between osteoarthrosis and osteoporosis groups.

Variable	Osteoarthrosis	Osteoporosis	*P* values*
ISQ fresh	73.53 ± 1.26	67.13 ± 1.09	0.002
ISQ fixed	72.33 ± 1.65	70.39 ± 1.61	0.406
ISQ decalcified	46.47 ± 4.34	51.03 ± 3.53	0.421

Values are expressed as mean ± standard deviation. *Bonferroni test.
